# Supplementary material for: Acidifiers Attenuate Diquat-Induced Oxidative Stress and Inflammatory Responses by Regulating NF-κB/MAPK/COX-2 Pathways in IPEC-J2 Cells
Source: Antioxidants (Basel). 2022 Oct 10;11(10):2002. doi: 10.3390/antiox11102002 (PMC9598074; doi:10.3390/antiox11102002)
Supplement: Supplementary file 1 [file antioxidants-11-02002-s001.zip › antioxidants-1916056-supplementary.pdf]

Table S1 Primer sequences used for real-time quantitative PCR.

Gene name	Accession number	Primer Sequence (5´-3´)
Claudin-1	NM_001244539.1	AGATTTACTCCTACGCTGGT<br>GCACCTCATCATCTTCCAT
Occludin	NM_001163647.2	ATGCTTTCTCAGCCAGCGTA<br>AAGGTTCCATAGCCTCTCGGTC
ZO-1	NM_005659811.1	GAGGATGGTCACCGTGGT<br>GGAGGATGCTGTTGTCTCGG
TNF-α	NM_214022.1	CCACGCTCTTCTGCCTACTGC<br>GCTGTCCCTCGGCTTTGAC
IL-8	NM_213867	TAGGACCAGAGCCAGGAAGA<br>AGCAGGAAAACTGCCAAGAA
IL-10	NM_214041.1	ACCAGATGGGCGACTTGTTG<br>TCTCTGCCTTCGGCATTACG
BAX	XM_003127290.5	CCGAAATGTTTGCTGACG<br>AGCCGATCTCGAAGGAAGT
BCL-2	XM_001928880.6	TTCTTTGAGTTCGGTGGGG<br>CCAGGAGAAATCAAATAGAGGC
PCNA	NM_001291925.1	AATGTTGATAAAGAGGAGGA<br>TAGGAGAGAGTGGAGTGGCT
CCND1	XM_021082686.1	CAGAAGTGCGAGGAGGAGGT<br>CGGATGGAGTTGTCGGTGTA
COX-2	NM_214321.1	AGAAGCGAGGACCAGCTTTC<br>AAAGCGGAGGTGTTCAGGAG
NF-κB	NM_001048232.1	CTTACACTTGGCAATCATCC<br>ATAGCGTTCAGACCTTCAC
I-κB-α	NM_010907.2	TGCAGGCCACCAACTACAAT<br>TCAACAAGAGCGACACCAGG
I-κB-β	NM_001099935.1	CTGCCTGTCCAAGATGAAGAACTCC<br>GTCCGATGTGATCCCAAACTCTGTC
ERK1	XM_021088019.1	CCGCTTGCCTCATTAAAGCC<br>TTAGACGTGGCAGCTTGGTT
JNK2	XM_005661441.3	AAACAAGTCAAGCCAGAG<br>AGCAGGGTCATACCAAAC
GAPDH	NM_001206359.1	CGGAGTGAACGGATTTGGC<br>CACCCCATTTGATGTTGGCG
